# Abstracts and Gleanings

**Published:** 1878-07-20

**Authors:** 


### *Abstracts and Gleanings.*

### THE METRIC SYSTEM IN A NUT SHELL.

"Universality, Uniformity, Precision, Significance, Brevity, and Completeness. A system of weights and measures born of philosophy rather than of chance."

BY EDWARD WIGGLESWORTH, M.D.

WASHINGTON, May 3.—Surgeon-General Woodworth, of the United States Marine Hospital Service, has issued a circular, with the approval of Secretary Sherman, requiring medical officers of the Marine Hospital Service to make use hereafter, for all official, medical, and pharmaceutical purposes, of the metric system of weights and measures, which had already, under the act of July 28, 1866, been adopted by this service for the purveying of medical supplies."—*Boston Daily Advertiser, May* 4, 1878.

The metric system is already legalized in both America and England. The only question now is, which of the two, the most progressive or the most conservative nation on earth, shall be the first to definitely and finally adopt it as an exclusive system? [N. B.—England was 400 years behind the continent in adopting our present arithmetic.] Russia has already taken the preliminary steps towards its final adoption. The rest of the civilized world long since made the system obligatory, in whole or in part, except that, in Sweden alone, its obligatory use is to date from a period in the future, 1889.

Now, what is this metric system? Metric is from the Greek word, *metron*, a measure, spelled with Epsilon, e short, and, therefore, pronounced met-ric.

The meter (measure) is, practically, a fixed quantity, namely, the ten millionth part of the earth's quadrant from the equator to the north pole. With the meter, everything can be measured, for it is itself the unit of length; a cube, the edge of which is the tenth of a meter, is the unit of capacity (liter), and the weight of a cube of rain water, at its extreme contraction, the edge of which cube is a hundredth of a meter, is the unit of weight (gram).

It is the gram alone which concerns physicians, for, in the metric system, everything is best prescribed and dispensed by weight alone—numbers upon a prescription paper being regarded by the pharmacist as representing grams, unless the contrary is expressly stated. The fractions are always decimal.

The table is easily learned. It consists of six words, as prefixes, whether we deal with grams, liters or meters. These are: deci for tenth, centi for hundredth, milli for thousandth; deka for ten, hekto for hundred, kilo for thousand. Having these few words, the terms of troy, avoirdupois, and apothecaries' weight, and of liquid measure, may be relegated to the limbo of pounds sterling, shillings, four-pence-ha'pennies, and farthings. As we say dime, cent, mill, so we say decigram, centigram, milligram. These prefixes are Latin, and diminish the value. Deka, hekto, and kilo are Greek, and increase the value. The mnemonic is G I L D, i. e., Greek increases, Latin decreases. Deka occurs in the English word decade; hekto in hecatomb; kilo in chiliad.

"Being accustomed to the words mill, cent, and dime, we shall find the words milligram, centigram, and decigram quite as simple and easy to pronounce as our words pennyweight-troy, hundredweight-avoirdupois, scruple-apothecaries, etc., notwithstanding the assertion to the contrary of those who grieve to give up the short and sharp Anglo-Saxon words used in our present familiar old tables of weights and measures."

Practically, moreover, for physicians, the whole system is reduced to grams and centigrams, just as, in money, to dollars and cents. On the right side of the prescription paper, draw a perpendicular line from top to bottom. This decimal line takes the place of all the decimal points, and obviates the possibility of mistakes. This is the way dollars and cents are separated on business papers. Additional security is gained by writing the decimal fraction (centigrams) of half size, and raised above the line (of grams), since it represents a numerator of which the denominator, 100, is omitted. To make assurance doubly sure, "grams" may be written over the integer-column of figures, and, if wished, the word "decimal" over the decimal column.

Now, what is a gram? or rather the values, metrically expressed, of our present awkward weights?

| | Prussian | Practical | Precise |
|---|---|---|---|
| Grain I = | 0.06 | 0.06 | 0.065 |
| Scruple I = | 1.25 | 1.25 | 1.29 |
| Drachm I = | 3.75 | 4.0 | 3.89 |
| Oz. I = | 30.0 | 32.0 | 31.1 |

The "practical" table alone concerns us. The "Prussian" (by order of the Prussian Ministry, Aug. 29, 1867) is given merely to show that our table is even nearer the actual truth than one which has been proved by actual experience to answer every purpose. The values of the grain and scruple are a little too samll. As they are used for yowerful drugs this is an error in the right direction. The values of the drachm and ounce are a trifle too large, but the proportions and therefore the ratio of drug to vehicle are preserved.

A prescription written metrically is always proportionate, and whether the pharmacist uses pennyweights, pounds, or tons; gills, pecks, or chaldrons; pints gallons, or hogsheads, the ratios are preserved, and a teaspoon dose contains the same amount of medicine.

As regards administration, a teaspoon represents five grams; a tablespoon twenty grams; for a teaspoon holds one and one-third fluid drachms, a tablespoon a trifle more than four times as much.

In the metric system *everything is weighed*, thus obviating the difficulties of evaporation, refraction and adhesion, and obtaining more conveniently, more exact results. In our old "systemless system" some fluids were measured. How shall we obtain with weights, the desired bulks of fluids vith varying weights? Must we learn the specific gravity of all fluids?

*Not at all!*

1. Fixed oils, honey, liquid acids and chloroform, must at present be prescribed in our old weights, not measures, according to the pharmacopœia. Here change old weights to metric ones.

2. Not enough chloroform or ether is included in any one prescription to admit of harm arising from the amount contained in a single dose, even were their weights regarded as the same with that of water. Moreover, it is not difficult to remember that ether weighs seven-tenths as much as water, chloroform twice as much as ether.

3. There remain infusions and tinctures, glycerines and syrups. These four are used in bulk as doses, or as solvents or vehicles. The former two may be regarded as identical in weight with water; the latter two as one-third heavier, and when prescribing these we need merely write, by weight, four-thirds as much as we should write for were we prescribing water, and we obtain an equal bulk. the teaspoon or tablespoon dose will then contain the desired amount of the drugs employed.

Or, simplest of all, we can make any mixture up to any desired bulk by merely directing the druggist to use enough of the vehicle to bring the whole mixture up to the requisite weight for that bulk.

The Metric Bureau, 32 Hawley street, Boston, will furnish metric prescription-blanks to order, to druggists or physicians at four-fifths printer's rates, or any blank can be made sufficiently metric by a perpendicular line at the right, headed *Grams.*

### DISEASES OF THE EYE.

From the nature and condition of the different organs that enter into the formation of the eye, it is utterly impossible for any one nostrum to meet ail the requirements of the various diseases. Even the same disease may require a change of treatment.

There are some diseases of the eye, during the course of which it is absolutely necessary for the patient to be under daily observation. But a great many people suffering from chronic conjunctivitis or granulated eye-lids, can be examined occasionally, and remain at home the rest of the time. We do not make this statement as a mere hypothesis, but speak from experience.

CASE I —CHRONIC CONJUNCTIVITIS.

John Hale, æt., 28. Has never had any ophthalmic trouble previous to the present attack. About a year ago he noticed that when the wind blew into the right eye, it felt weak and would weep freely. This did not give him very much trouble at first, but later he noticed that the weeping became more marked, so much so at times he had to protect it with his hand, or keep it closed. He did not have any pain in it, neither did the light produce any unpleasantness, but simply the inconvenience and annoyance from the free secretion of tears. A few weeks later he noticed a small amount of muco-purulent matter at the inner corner of the eye, of a morning. He did not have any uncomfortable sensation in or about the eye, such as grit or fine sand under the lids. A few of the veins in the occular conjunctiva were dilated. On everting the upper lid it was found that the inflamation was confined to that portion of the conjunctiva that corresponds to the upper margin and extremities of the tarsus. On the lower lid the inflammation was general, involving the entire conjunctiva.

The treatment in this case began with the usual astringent remedies used for conjunctivitis. [illegible] might have been all that was required, but in the majority of cases of chronic trouble, there is more or less debility of the constitution. In addition to the above he received constitutional treatment consisting of the chalybeate and bark preparations. Ferrum dialysatum is very good given in fifteen drop doses three times a day, after meals. Upon this course he made a good recovery, and has had no trouble with his eyes since.

CASE II. CHRONIC GRANULAR EYELIDS.

Clave Jones, æt. 33, German. Has been troubled with sore eyes for twelve years. Does not know any particular cause, but thinks it came from exposure during a rainy season. The trouble began as an ordinary "cold in the eyes." At first there way considerable redness and the eyes would weep freely when exposed to sunlight. This however passed off to a great extent in a few days, so that he did not consider it necessary to h ve them treated. He expected, that as they had improved so much during the first few days, that they would continue and be well in a short time. But there remained a small amount of matter at the corner of the lids. At first this was not larger in size than a pin's head, but in the course of a few weeks it increased to twice and three times that amount. His eyes, up to this time, had not pained him, but now they felt as if there was fine sand or grit under the lids. This was noticed first of mornings and would disappear as soon as he bathed them in cold water, and would feel first-rate the rest of the day. The next morning however, they would be in the same condition, until after the cold water had been used. If he was in the wind they would feel weak, and the tears would gather so fast that it affected his vision. After a while the light began to be painful, and he had to use eye-goggles. This last resort appeared to ease them for a while, but he soon found that his sight was failing. The scratching sensation under the lids was present during the entire day. When he kept his eyes closed and perfectly at rest he did not notice it. He used different kinds of eye lotions and was prescribed for by a number of physicians but received only temporary benefit. For the past three years he has done nothing for them, and had given up all hopes of the right eye.

The conjunctivæ of the lids were reddened and inflamed and presented a velvety appearance, in some places it was tendinous. The cornea of the right eye looked like a nebulous mass streaked with bloodvessels. There were a number of ulcers on the cornea. Light was very painfnl. Vision wrs very poor in this eye as he could only count fingers at a distance of six inches. Vision in the left eye was $\frac{10}{100}$. The lids were not contracted, and there did not appear to be any undue pressure upon the globe. The operation of syndectomy had been performed upon the right eye with no apparent benefit.

The treatment in this case was both constitutional and local. The first, I think as important in this case as the second, consisted of the following:

| | | | |
|---|---|---|---|
| R | Quin. sulph., | gr. | ss. |
| | Ferri redact, | gr. | j. |
| | Ext. tarax., | gr. | ss. |
| | Pic. liq., | gr. | j. M. |

Ft. pill.—Sig.: one after meals.

The local treatment consisted in the use of atropine and the mild astringents. The eyes were protected from the light with a shade. As he was a great smoker he was advised to indulge as little as possible, and to have the eyes well protected at such times. Smoking being an irritant is injurious to an inflamed eye. Atropine was used daily. Bathing the eyes in a solution of sulphate of zinc is very good in some cases and had a very good effect in this one. Under this treatment the eyes made a good recovery. Caustics were not used in any manner, as he had been through one or two courses of such treatment. He stated that one physician to whom he applied, used a solution that felt like live fire coals in the eye, the pain produced being almost intolerable.—*Lancet and Observer.*

ARM PRESENTATIONS.

Meadows, in his *Manual of Midwifery*, says, under the heading of "Presentation of the Upper Extremity," that little time should be lost in trying questionable expedients, for there is no better way known than to "turn and deliver by the feet." I believe this is an old axiom, and it is easily remembered. It may not be easy to call to mind every rule in an undetermined case, for arm presentations do not occur often enough to keep us familiar with every feature of the complication. It is not hard to tell, by the thumb, the fingers, and the palm, which extremity—the right or the left—is presenting, and then the problem is not exceedingly blind to determine which foot is to be grasped first in order to facilitate delivery.

Right here I venture to say that not one practitioner in a hundred knows just what to do in a complicated labor, unless he has had some experience with obstetric troubles. In most instances, labor is so simple and easy that the obstetrician is lulled into a state of mental security, which may prove disastrous to his reputation some pleasant day.

Presentation of the upper extremity occurs once in about two hundred and fifty labors; hence, a physician may go through many years of active practice and not meet with an arm presentation, and the first case may come quite early in professional experience.

Recently, I asked a physician, of fifteen years' practice, what was the extent of his obstetrical business. He replied that, during the first five years of professional duties, he had forty cases of midwifery. During the succeeding five years, he had sixty cases, and during the last five years, he had had one hundred cases. I then asked him if he had ever met with an arm presentation, and he said that he had not. Now, said I, your chance for trouble is near at hand; you have had two hundred cases of labor, and no arm presentation. An upper extremity presentation occurs once in less than two hundred and fifty cases. Ac-

Page(s) missing days to amuse herself. She went to the opera, and in going down the stairs caught her foot in the train of a lady's dress. She fell forward and struck her head slightly against the opposite wall. She felt a little giddy, and said that she would not go into the theatre, that she would return home. She went to bed, fell asleep, and about ten in the morning, when the maid came to wake her, she found her so fast asleep that she did not like to disturb her; but about twelve o'clock the friends got alarmed, and they sent for a neighboring medical man, and he came for me. I found her comatose, suffering from compression of the brain, and went home to get my trephines, but when I came back she was dead. A post-mortem examination was made, and we found a clot of blood the size of a small saucer on the side that was struck, between the skull and dura mater over the course of the middle meningeal artery, but without any fracture of the skull.

Some years ago a cabman was thrown off his box, and he became slowly comatose. Three days after the accident he was brought to the hospital. When I saw him he was suffering from a profound coma, and there was some paralysis of the side opposite to that on which he had been struck. I cut down upon the skull, and found a starred fracture in the right temporal bone. I trephined him, and found a large clot of blood under the bone. Some blood welled out rather freely, evidently from the middle meningeal artery. The flaps of scalp were laid down, and he made a very good recovery.

The explanation of these cases given by Sir Charles Bell many years ago showed experimentally how these extravasations are occasioned. He took a wooden mallet and struck a forcible blow upon the side of the head of a dead body in the dead-house. On removing the skull-cap he found that the dura mater was detached from the seat of the blow, although there was no fracture. He went further than this; he made the same experiment upon another subject, and after having made it he injected it with soft size. He injected this into the arteries, and found, after the size had been allowed to cool, that it had become extravasated, and had formed a large clot between the dura mater and the skull. There you get the exact condition of things that we meet with in the wards and operating theatre—namely, a separation between the dura mater and the skull, and an extravasation of blood between the dura mater and skull where they are separated. From these interesting observations it would appear that there are two distinct sources of hæmorrhage between the dura mater and skull. In the first case the middle meningeal artery is torn across by a fracture traveling across the anterior inferior angle of the parietal bone; and in the second case, in which the artery is not torn, but an accumulation takes place from the smaller branches that get torn at the time the shaking occurs which separates the dura mater from the skull, and which allows oozing to go on, and produces a slow supervention of coma—what you may call "surgical apoplexy." It has been supposed that the separation between the dura mater and skull was effected by the impulse of blood driven out from the torn middle meningeal artery which pushes away the dura mater from its connexions with the skull, and as it pushes away the dura mater the cavity so formed is filled with blood. Sir Charles Bell conclusively proved, by the experiment to which I have referred, that separation of the dura mater was the primary condition; and there can, I think, be little doubt that the detachment of the dura mater is the result of the blow on the head, and the filling is the consequence of that detachment, and that it could not take place if the detachment had previously occurred. The vacant place gradually gets filled up with blood, more rapidly if the trunk of the middle meningeal artery be torn across, when it will become full in the course of half or three-quarters of an hour after the accident. When the main trunk escapes, and it is only the terminal branches that get torn, you get that set of cases in which the accumulation of blood goes on much more slowly, and only compresses the brain to such an extent as to give rise to coma in the course of several hours.

---

THE MEDICAL TREATMENT OF CHILDREN.

According to Dr. Eustace Smith, of London, the alkalies are remedies of singular value in the medical treatment of young children. In all children, especially in infants, there is constant tendency to an acid fermentation of their food. This arises partly from the nature of their diet, into which milk and farinaceous matters enter so largely; partly from the peculiar activity of their mucous glands, which pour out an alkaline secretion in such large quantities. An excess of farinaceous food, therefore, soon begins to ferment, and an acid is generated, which stimulates the mucous membrane to further secretion. In all chronic diseases, and in many of the acute disorders, this sour condition of the stomach and bowels is present. Alkalies are therefore useful—firstly, in neutralizing the acid products of this fermentation; and, secondly, in checking the too abundant secretion from the mucous glands. A few grains of soda or potash, given an hour or two after taking food, will quickly remedy this derangement and remove the distressing symptoms which arise from it. In the chronic diseases, indeed, attention to this point is of especial importance; for by placing the stomach and bowels in a healthy state, and insuring a proper digestion of food, we put the child in a fair way of recovery, and prepare the way for the administration of tonic and strengthening medicines, by which his restoration to health is to be brought about.

In prescribing for infants, an aromatic should always be included in the mixture. The aromatics are useful, not only for their flavoring properties, but also for their value in all those cases of abdominal derangement where flatulence, pain, and spasm, resulting from vitiated secretions and undigested food, are present to increase the discomfort of the patient. Such dyspeptic phenomena are usually relieved rapidly by the use of these agents; and ainseed, cinnamon, caraway-seed, or even tincture of capsicum in minute doses, will be found important additions to the prescription in all cases where alkalies are required.

In prescribing for children, the proper dose of a medicine cannot always be calculated according to the age of the child, and does not in all cases bear the same proportion to the quantity suitable for an adult. For certain drugs, children show a remarkable tolerance, while to the action of others they show as remarkable a susceptibility. Thus, opium, it is well known, acts upon a child more powerfully than would be expected, judging from the mere difference of age. It should therefore be given to infants with a certain caution, especially if the child be enfeebled by disease. It is, however, a medicine which is of especial value in the treatment of the diseases of infancy, and may be given without fear if care be taken not to repeat the dose too frequently. Belladona, on the contrary can be taken by children in larger quantities. A child of two or three years will bear without inconvenience a dose which in an adult might produce very uncomfortable symptoms. It is important to remember this in giving belladona for its sedative effects, as in whooping-cough. Lobelia, again, is a remedy which is very well borne by children. Dr. Ringer has given it to "very young children," in doses of five minims every hour, and in no case has he noticed any ill effects to follow its administration. Arsenic should be given to children over five years of age in the same dose as that used for adults, and infants a month or two old will take one drop of Fowler's solution three times a day with great benefit in case of gastric catarrh. The influence of mercury upon young children deserves remark. It seldom in them produces stomatitis or

Pages 10. 11. 14. 15 wanted
duplicate pages cut out.
caused by pressman
backing the sig wrong.

Binder

Page(s) missing was introduced into the uterus. Pains came on in three or four hours, and the patient was delivered of a vigorous male child; but in forty-eight hours puerperal septicæmia came on and proved fatal. Perhaps a better result, even in this case, would have followed earlier induction of labour. One month after, the lady's sister, in the same house, attended by Dr. Sands, after a perfectly natural labour, died of puerperal fever.—*London Lancet.*

---

MATE OR PARAGUAY TEA.

*Phamaceutical Preparations.*—The following preparations of mate are suggested: The simple infusion which is the form in which it is always used in South America; a solid extract prepared with alcohol of sp. grav. ·822, and a fluid extract prepared with alcohol of sp. grav. ·941, in such proportion that when finished its weight will be equal to the weight of mate used in its preparation. A considerable quantity of fluid extract prepared by this formula has been used in debility and in various derangements of the nervous system, generally with satisfactory results.

The reputed therapeutical properties of mate have been fully stated in a number of heretofore published papers, some attributing the most deleterious effects to its continued use, and others lauding it to the utmost limit of credibility, almost equaling the marvelous statements made of the action of the somewhat similar substance, Coca. In regard to mate, however, the writer is fully convinced that it does really possess properties which render it worthy of careful therapeutical investigation.

The thorough desiccation it undergoes in its preparation, and the compact and hermetical character of the packages in which it is contained, tend greatly to the preservation of whatever virtues it may have originally possessed.—*American Journal of Pharmacy.*

---

THE MICROPHONE IN MEDICINE.

We find on the one hand Dr. Richardson of London, examining and noting the heart-and-lung sounds with the aid of the microphone, and, on the other, Sir Henry Thompson lecturing on the use of the microphone in searching for stone and in probing for bullets or for diseased bone. When we remember that by means of this instrument the crawling of a fly over a piece of gauze may be rendered as audible as the tramp of an army, or its breathing as distinct as the bellowing of the leviathian, we can already look forward to treatises on the sounds of inflammation and the rythm of fevers; the harmonies of health and the discords of disease will no longer be fanciful similes, but scientific facts, and the poet's assertion that "there is in souls a sympathy with sounds" will be philosophically verified.—*Medical Times.*

---

NEW PLAN OF TYING KNOTS IN SURGICAL OPERATIONS.

Dr. Ewell, Jr., in Virginia *Medical Monthly* says:

I have been much annoyed in tying ligatures and sutures by the first part of the knot slipping while I was tying the second part.

To remedy this we commonly press upon the first part of the knot with the finger or probe, or hold it with a pair of forceps while we form and secure the second part. But this requires the aid of an assistant, who is not always on hand; besides, I think a surgeon should not require help in so simple an operation as tying a knot. I find that if, when making the first part of the knot, I pass one cord under the other *two or three times* instead of *only once*, this, the first part of my knot, will not slip while I am making the second part. To this may be added a third part to make it more secure.

I have never seen any mention of this plan in surgical works, and if such exists I am ignorant of it. As I have never seen this method used, except when I advise it, I think it might be new to some of the readers of the *Virginia Medical Monthly.* A knowledge of these little points go far to make up the neat operator, and it is of special comfort to the country practitioner so frequently alone at his operations.

BROMIDE OF ARSENIC IN THE TREATMENT OF NERVOUS DISEASES.

Clemens (Allg. Med. Cent. Zeitung) states that he has obtained astonishing results with bromide of arsenic in the treatment of diseases of the nervous system, and especially epilepsy. The following is the formula which he thinks should replace Fowler's solution:

℞ Pulv. arsenic alb.... } aa oz i;

Potass. carb.......... }

Coque cum aq. dest..... ℔. ss;

Ad solut. perfect; aq. evaporat. adde—

Aq. dest.............. oz xij;

Brom. pur............ dr ii.

Refrigerat. Of this he gives one or two drops in a glass of water once or twice daily. This dosage may be continued for months, or even years, without producing any unpleasant effects. In only two cases of epilepsy did he effect a complete cure, but in all the cases marked relief was obtained. In connection with the bromide of arsenic an almost exclusively meat-diet is advised. The patients should be as much as possible in the open air. Unlike the bromide of potassium, the arsenical salt does not require to be given in increasing doses, and, instead of interfering with digestion, improves the nutrition and strength.—*The Doctor.*

DATURIA AS A MYDRIATIC.

Jobert (de Lamballe) proposed, as early as 1861, to substitute daturia for atropia as a mydriatic, and an analysis of his work was published in the *Bull. Gen. de Therap.*, Vol. lxii. His conclusions in reference to its properties were: 1. Daturia is three times as active as atropia and its salts; therefore, its dose should be only a third as great as that of the preparation of atropia. 2. When introduced between the lids, it does not cause the pain and confusion of vision which attend the similar use of belladonna. 3. The effects of daturia are more constant than those of belladonna, and its action persists for a longer time than that of the latter.—*Bull. Gen. de Therap.*

ACTION OF STRYCHNIA ON THE EYE.

Dr. Hippel says that he has found, by personal experience, that, when given in doses of two to four milligrammes, strychnia produces the following effects, namely: 1. Increased peripheric sensibility for blue. 2. Temporary increase of visual power. 3. More distinct perception of peripheric points. 4. Lasting enlargement of the field of vision. He believes that the effect on the optic nerve is the same as that attributed to continuous electric currents on other nerves.—*New Remedies.*

MURIATE OF AMMONIA FOR THE HYDROCELE OF INFANTS.

Saint Germain believes that it is not advisable to subject an infant, with hydrocele, to even the simplest operation, until a trial has been made of a saturated solution of muriate of ammonia. Compresses dipped in such a solution should be applied. Sometimes an erythema, even slight vessication may be caused, but the part may be covered with powder, and the cure is not retarded.—*Am. Practitioner.*

POST PARTUM HEMORRHAGE—NEW METHOD OF USING PERCHLORIDE OF IRON.

Dr. James Brisbane (London *Lancet*), in cases of post partum hemorrhage, applies to the bleeding surface of the uterus a sponge soaked with tincture of iron. The blood coagulates, the uterus contracts, and the patient is out of immediate danger. At the following visit, the sponge is found in the vagina. All the apparatus needed is a two-ounce vial of tincture of iron and a sponge. In all the cases thus treated—four—the results were all that could be desired.—*Maryland Med. Jour.*

